# Extracellular Influences: Molecular Subclasses and the Microenvironment in Pancreatic Cancer

**DOI:** 10.3390/cancers10020034

**Published:** 2018-01-27

**Authors:** Veronique L. Veenstra, Andrea Garcia-Garijo, Hanneke W. van Laarhoven, Maarten F. Bijlsma

**Affiliations:** 1 Laboratory for Experimental Oncology and Radiobiology, Center for Experimental and Molecular Medicine, Academic Medical Center and Cancer Center Amsterdam, Meibergdreef 9, 1105 AZ Amsterdam, The Netherlands; andreagarciagarijo4@gmail.com (A.G.-G.); m.f.bijlsma@amc.uva.nl (M.F.B.); 2 Department of Medical Oncology, Academic Medical Center, University of Amsterdam, Meibergdreef 9, 1105 AZ Amsterdam, The Netherlands; h.vanlaarhoven@amc.uva.nl

**Keywords:** pancreatic cancer, heterogeneity, stroma, immune cells, fibroblasts, mutations, gene expression

## Abstract

Pancreatic ductal adenocarcinoma (PDAC) is the most prevalent form of pancreatic cancer and carries the worst prognosis of all common cancers. Five-year survival rates have not surpassed 6% for some decades and this lack of improvement in outcome urges a better understanding of the PDAC-specific features which contribute to this poor result. One of the most defining features of PDAC known to contribute to its progression is the abundance of non-tumor cells and material collectively known as the stroma. It is now well recognized that the different non-cancer cell types, signalling molecules, and mechanical properties within a tumor can have both tumor-promoting as well as –inhibitory effects. However, the net effect of this intratumour heterogeneity is not well understood. Heterogeneity in the stromal makeup between patients is even less well established. Such intertumour heterogeneity is likely to be affected by the relative contributions of individual stromal constituents, but how these contributions exactly relate to existing classifications that demarcate intertumour heterogeneity in PDAC is not fully known. In this review, we give an overview of the available evidence by delineating the elements of the PDAC stroma and their contribution to tumour growth. We do so by interpreting the heterogeneity at the gene expression level in PDAC, and how stromal elements contribute to, or interconnect, with this.

## 1. Introduction

The most common form of pancreatic cancer (PC) is pancreatic ductal adenocarcinoma (PDAC), which represents over 85% of all PC cases. PDAC is ranked as the fourth leading cause of cancer-related deaths, with a median 5-year survival rate of 3% in Europe [1,2,3,4]. Its aggressiveness, resistance to chemotherapy, and late detection, contribute to the poor prognosis of this disease. Only 20% of the patients are eligible for surgery, which is currently the only treatment with a truly curative intent [2].

On a histological level, one of the most remarkable features of PDAC is a dense stroma. The stroma can constitute up to 90% of the tumour bulk and is characterized by extensive fibrosis, lack of vascularization, immune infiltrates, and hypoxia [5]. This fibrotic tissue is formed by non-cellular and cellular components, consisting of cancer-associated fibroblasts (CAFs), which mostly derive from pancreatic stellate cells (PSCs) [6]. The extracellular matrix (ECM) is the principal non-cellular component of the fibrotic stroma, and is composed of collagens, glycosaminoglycans, proteoglycans, and growth factors [7,8]. This process of excessive deposition of connective tissue is also known by the medical term desmoplasia.

The interaction between stromal and pancreatic cancer cells that together form the tumour microenvironment (TME) plays a crucial role in PDAC development and progression. Two distinct stroma subtypes have been identified that are independent of tumour subtype and impact on patient survival [9]. Therefore, identifying the aberrant activation of genes, proteins or cells specifically in the TME of PDAC could potentially give insight into the molecular biology of the subtypes in pancreatic cancer. In this review, we describe the development of PDAC and the factors that are involved in this development. Mainly we aim to delineate the components of the TME in PDAC—the extracellular matrix, vascularization, cancer associated fibroblasts and immune cells—describe their function, explain how the tumour cells have found a way to use these components for their own benefit, and discuss how this can contribute to tumour bulk heterogeneity.

## 2. Development of PDAC

The development of PDAC is thought to be a sequential process that relies on the accumulation of mutations. PDAC tumours can evolve from pre-malignant precursor lesions; mucinous cystic neoplasms (MCN), intraductal papillary mucinous neoplasms (IPMN) and pancreatic intraepithelial neoplasia (PanIN). PanINs are the most common and widely studied precursor lesions. These lesions are classified into three grades based on their morphology, ranging from low grade PanIN-1A/PanIN1B to advanced grade PanIN3s [10]. Elemental to the progression of these PanINs is the accumulation of genetic aberrations, commonly starting with an activating mutation in KRAS, after which additional mutations in CDKN2A, TP53 and SMAD4 are common [11,12,13]. It should be noted that this concept of gradual mutational burden was recently challenged; it has been suggested that mutations occur in a relatively rapid sequence of large genetic events [14].

### 2.1. Mutational Status of PDAC

Activating mutations in KRAS are a hallmark of PDAC, and are found in nearly 95% of cases [15,16]. The frequent occurrence of KRAS mutations in PDAC was already observed before large-scale next generation sequencing efforts [17,18]. The major downstream effectors of KRAS regulate proliferation, survival, migration, and invasion in PDAC [19]. Despite being accepted as an initiating event for PDAC, oncogenic KRAS by itself is insufficient to drive PDAC. Recent efforts to report the complete genetic profile of PDAC revealed a complex mutational landscape [15,16,20]. Besides inactivating KRAS mutations, over 50% of all mutations found affected the CDKN2A, TP53 and SMAD4 genes. Other common mutations make up 10 percent of mutated genes, and there is a large group of infrequently mutated genes that is suspected to contribute to the inter- and intra-tumour heterogeneity. 

Whole genome sequencing and copy number variation (CNV) analyses have been used to classify PDAC into four subtypes by patterns of structural genetic variation. These 4 subtypes range from stable, locally rearranged, scattered, to an unstable subtype, all referring to the degree of structural variations present in the genome [21]. At a low individual prevalence (1–2% of patients), some common amplifications in the locally rearranged subtype are therapeutically targetable, like ERBB2, MET, CDK6, PIK3CA, and PIK3R3. The unstable subtype was accompanied by inactivation of DNA maintenance genes, like BRCA1, BRCA2 and PALB2, which are also amenable to targeting. Studies that aim to implement genetic tumour profiling of patients in the treatment decisions have thus far been largely unsuccessful [22,23]. The IMPaCT trial did not treat any of 22 eligible patients included, and a Phase I clinical trial program at MD Anderson treated 1 out of 34 eligible patients with matched therapy [23]. Actionable targets however are present in 26–35% of samples analysed in larger archival or prospectively collected samples [24,25]. Progress is being made to tackle the challenges that exist in the acquisition of tumour samples for genetic profiling, however this strategy is not yet ready for implementation in the clinical routine.

### 2.2. Signalling Pathways in PDAC

Several signalling pathways in PDAC contribute to the development and maintenance of the stroma. One of the most prominent is the role of the Hedgehog (Hh) pathway in PDAC progression, as was demonstrated in 2003 [26]. Later, it was found that Hh signals to the stroma rather than the tumour cells, and there it activates the secretion of ECM proteins and the production of pro-tumorigenic factors, beneficial for the tumour cells [27,28,29]. Proteins upregulated in the stroma in response to Hh signaling are associated with an increased proliferation and survival of pancreatic cancer [30]. Another important pathway in pancreatic cancer is that of the transforming growth factor β (TGF-β) ligand [16]. The common loss of SMAD4 in PDAC results in the upregulation of TGF-β signalling, which aids in tumour progression via the activation of MAPK/ERK, PI3K/AKT, p38 MAPK and Rho-GTPase pathway signalling [31].

**Figure 1:**
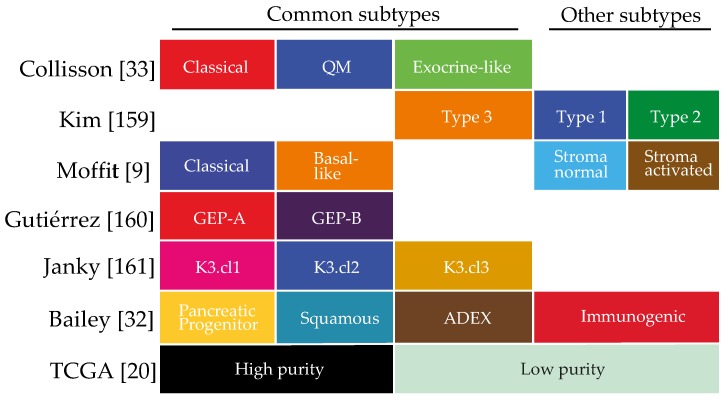
Schematic overview of all reported expression-based subtypes in PDAC. Correlated subtypes are grouped in columns. The “Other subtypes” could not be grouped. Colours are based on the colour coding used in the original publications.

The sum of the complex contributions of the many deregulated genetic, epigenetic and signalling pathways in PDAC, as well as any extracellular influences, can be best captured at the mRNA level. Unsupervised classification methods of the transcriptome have aimed to classify pancreatic cancer based on their expression patterns [9,20,32,33]. Several of such classifications have been reported and although the number of subtypes identified differs, all share subtypes that are apparently consistently present in pancreatic cancer (Figure 1). For instance, all classifications identify a canonical/classical subtype, characterized by epithelial genes, and a mesenchymal-like/poor-prognosis subtype, characterized by expression of mesenchymal genes [34]. Subtypes that relate to the exocrine functions of the pancreas have been identified, as are immunological subtypes characterized by expression of immune cell-related genes. These classifications are mostly tumour based but are obviously affected by the contributions of stromal gene expression as well.

### 2.3. Tumour Heterogeneity

These subtypes are the result of inter- and intra-tumour heterogeneity, and are the sum of differences in morphology, cellular composition, genetic makeup, and many other levels of biological information that exist between or within cancers, respectively. This heterogeneity can have consequences for the diagnosis of a tumour, as well as for the treatment. The onset of intra-tumour heterogeneity for tumour cells is thought to evolve from a cancer stem cell model or a clonal evolution model [35]. The cancer stem cell model assumes that there is one population of cells that bears true tumorigenic potential (i.e., to be able to populate the tumour), and has the ability to proliferate and differentiate into non-tumorigenic offspring. This differentiation is thought to create heterogeneity found within tumours. The clonal evolution model asserts the possibility of multiple cellular clones to be tumorigenic, these clones originate from acquired additional oncogenic mutations during proliferation. This expansion of clones is thought to also contribute to inter-tumour heterogeneity.

These models are typically tested in tumour cell models only, and the impact of the stroma is normally not taken into account. In order to take the stromal contributions into consideration we must know the constituents of the stroma.

## 3. Stromal Components: The Extracellular Matrix

The ECM normally functions to maintain tissue integrity by providing structural and biochemical support to cells. The ECM is constantly remodelled by enzymes like matrix metalloproteases (MMPs) and tissue inhibitor metalloproteinases (TIMPs). The ECM can be modified in abundance and composition, and this is important for example in wound healing. In PDAC both the pancreatic cancer cells and the stromal cells act on the ECM by the secretion of structural components and modifying enzymes [36,37]. In highly fibrotic tumours like PDAC, pancreatic cancer cell survival and ECM constituents are strongly interconnected and have a big impact on tumour cell motility.

### 3.1. Collagens

The main component of the ECM is collagen I, which promotes cell adhesion, proliferation and increased migration via α2β1 integrins by disrupting cell-cell adhesion contacts in normal tissue [38,39]. The production of collagen is stimulated by TGF-β and epidermal growth factor (EGF) signalling.

Decreased expression of E-cadherin in tumour cells is initiated by collagen I signalling via the upregulation of Smad-interacting protein 1 and activation of focal adhesion kinase (FAK) [40,41]. The disruption of these E-cadherin/β-catenin complexes force tumour cells into an accelerated cell cycle. Collagen I signalling also promotes n-cadherin expression by the activation of c-Jun NH2-terminal kinase signalling [42]. Collagen I signalling via the DDR2 receptor translocates NF-κβ to the nucleus and subsequently upregulates the expression of *snail* and *LEF-1* [43,44]. All these mechanisms are known mediators of epithelial-to mesenchymal transition (EMT) and promote tumour cell motility. Another ECM component that is involved in the carcinogenesis of PDAC is collagen V, which mediates trans-signalling via the α2β1 integrin as well. Collagen V promotes increased adhesion, proliferation and migration of PSCs, and it induces angiogenesis in the TME to support a metastasizing phenotype of tumour cells [45].

### 3.2. Laminin and Fibronectin

Laminins are heterotrimeric glycoproteins that consist of three chains, the α-, β- and γ-chain. They contribute to cell attachment and differentiation, mainly via integrin receptors [46]. Fibronectin is a glycoprotein in the ECM and is secreted by stellate cells, forming soluble protein dimers. Fibronectin mainly binds to integrin receptors and collagens inducing cell adhesion, migration and differentiation.

Detachment of the ECM in normal tissue induces apoptosis by cytochrome-c release and subsequent caspase activation. Laminin and fibronectin both contribute to the resistance against this cytochrome-c induced apoptosis and increase PDAC tumour cell survival by inhibiting caspase activity [47]. Besides the protection of tumour cell death, angiogenesis is also stimulated indirectly by fibronectin. Interleukin-8 secretion is increased upon fibronectin signalling, enhancing proliferation and invasion of endothelial cells [48,49].

### 3.3. Proteoglycans

Proteoglycans are small heavily glycosylated proteins, and function to bind ECM components like collagens. Proteoglycans can be expressed by tumour cells as well as stellate cells. Glypican-1 is a proteoglycan overexpressed in PDAC tumour cells, involved in tumorigenicity [50]. Exosome bound glypican-1 is also currently being investigated as a biomarker to detect early pancreatic cancer, emphasizing the importance of ECM components [51]. Another proteoglycan, expressed by the stromal compartment in response to Sonic hedgehog (SHH) signalling is SPOCK-1 [30]. SPOCK-1 is able to remodel the ECM, and subsequently indirectly enables tumour cells to become more invasive [52]. Lumican is a proteoglycan that is expressed in both the tumour as the stromal compartment, and directly interacts with tumour cells to inhibit tumour growth by inducing tumour cell quiescence [53].

### 3.4. Hyaluronic Acid

Hyaluronic acid (HA) is a glycosaminoglycan. It is formed in the plasma membrane instead of the Golgi membranes, allowing for large HA chains to be formed [54]. The molecular weight of HA is important for its signalling output; low molecular weight HA is associated with higher motility of tumour cells [55]. HA is formed by HA synthases (HAS), HAS-2 and -3 stimulate tumour growth when overexpressed in pancreatic cancer xenograft mouse models [56]. The main receptors of extracellular HA are the cell surface receptors CD44 and RHAMM. HA mediated signalling via CD44 or RHAMM induces a wide range of processes involved in cell adhesion, migration, invasion, survival and proliferation [57].

### 3.5. ECM-Targeting

The ECM has been found to reduce the efficacy of chemotherapeutic agents against tumour cells in vitro [58,59]. The desmoplastic reaction culminates a stiffened ECM, this stiffening of the ECM forces tumour cells into more chemoresistant cells and gain increased tumorigenic potential [60]. Laminin exerts this effect via the phosphorylation of FAK, suggesting efficacy of selective FAK dephosphorylating agents in terms of chemosensitivity [61]. Other targeting efforts could be focused on integrins, as most signalling between collagen, laminin and fibronectin is mediated by integrins. In line with this, the α5 integrin is shown to be the mediator of adhesion molecule L1CAM mediated chemoresistance [62]. When comparing 2D and 3D culturing methods in relation to chemoresistance, 3D culturing methods are generally more chemoresistant. The increased presence of ECM proteins in 3D cultures is thought to support this chemoresistance, via cell adhesion mediated drug resistance (CAM-DR) [59].

Clinical trials targeting ECM proteins have shown promising results: Combining hyaluronidase (HA degrading enzyme) to gemcitabine increases the overall survival of patients compared to single treatment, though this treatment was only beneficial in patients with high levels of HA [63]. An indirect method to target the ECM is via ROCK-1 and -2. ROCK-1 and -2 regulate the contraction of the actomyosin cytoskeleton and subsequent cell motility. ROCK is overexpressed in PDAC and exerts its effect on metastases via collagen remodelling in the ECM [64]. Short priming with ROCK inhibitors has shown promising effects with chemotherapy in pre-clinical models [65]. These therapies look promising, but they are based on the assumption that the ECM is pro-tumorigenic, and as we start to discover the duality of the stromal contributions to PDAC, this also pertains to the ECM.

## 4. Stromal Component: The Vasculature

The normal vasculature is relatively quiescent, and the endothelial cells (ECs) that line blood vessels only divide about once every couple years [66]. Blood vessels are not only made out of ECs, but also include pericytes and vascular smooth muscle cells (vSMCs). Without pericytes or vSMCs, blood vessels are immature and require vascular endothelial growth factor (VEGF) stimulation for survival [67]. The vascularization in PDAC is poor, which is not only due to vascular modulation via VEGF but also due to a high interstitial pressure. This results from the abundant and dense stroma that compresses existing capillaries, and leads to hypoxia and nutrient depletion [68]. It is important to look into the regulation of vascularization and the nutrient supply as for example in response to hypoxia, tumour cells undergo adaptive changes that allow them to survive and proliferate [69,70].

### 4.1. Angiogenesis

The formation of new blood vessels is called angiogenesis. The secretion of angiogenic factors and proteolytic enzymes in the TME ensures the formation of new blood vessels. Key regulators of the initiation of angiogenesis are cytokines, growth factors and hypoxia. The regulation of angiogenesis is unclear and very complex in pancreatic tumours. Many pro- and anti-angiogenic factors are produced in the TME.

Various cell types in cancer tissue such as tumour cells, PSCs, and infiltrated immune cells respond to hypoxia by the secretion of factors that act on angiogenesis. For instance, pancreatic cancer cells secrete large amounts of the anti-angiogenic endostatin in response to hypoxia, further contributing to a hypoxic environment [71]. In contrast, PSCs secrete high levels of VEGF, basic fibroblast growth factor (b-FGF) and periostin, all pro-angiogenic factors [72]. Sustained activation of PSCs also implies a constant deposition of ECM, restricting vascularization.

The effect of PSCs on angiogenesis is heterogeneous within tumours, as we can conclude from the fact that the pan- and juxtatumoral-stroma show a difference in vascularization. The juxtatumoral-stroma is the stroma in close proximity to the tumour cells mostly defined as the <100 µm from the tumour cells, whereas the pan-stroma comprises the rest of the stroma. The juxtatumoral stroma is hypovascularized due to tumour cell-secreted endostatin whereas the pan stroma, often associated with the invasive front of a tumour, presents a higher density of blood vessels, as was shown by the analysis of tissue microarrays [73]. In vitro and in vivo studies showed that infiltrating immune cells like macrophages promote angiogenesis by secretion of VEGF and MMP9. The degradation of the ECM by MMP9 modulates the mechanical stress on endothelial cells, enhancing endothelial cell migration and proliferation [74].

### 4.2. Nutrient Depletion

The lack of vascularization not only deprives the tumour of oxygen but also nutrients, causing metabolic stress. The metabolism of a tumour cell needs to be altered, as other nutrient sources need to be used. KRAS mutated cells are able to survive metabolic stress for instance by the uptake of extracellular proteins and lipids by scavenging [75]. Another mechanism that KRAS mutated cells employ is macropinocytosis, the internalization of proteins to serve as nutrient source [76]. Further nutrient sources come via the uptake of collagen I- and V-derived proline from the ECM, for entry in the tricarboxylic acid (TCA) cycle or glutamine synthesis [77]. Deprivation of nutrients is also known to induce autophagy, not only in cancer cells but more recently also discovered in PSCs. This autophagy of PSCs facilitates as a nutrient source for the cancer cells by providing them with non-essential amino acids (NEAA) such as alanine [78,79]. Further adaptation for nutrient sources can develop via mature endothelial cells. These cells form basal microvilli to increase cell surface area for nutrient exchange, and this results in increased glucose uptake in tumour cells [80]. When cells are severely deprived of essential nutrients like glucose, they can undergo changes that affect integrin intracellular trafficking, resulting in a decreased invasive migratory capacity [81].

### 4.3. Hypoxia

The cellular response to severe hypoxic conditions is mediated by hypoxia inducible factor 1α (HIF-1α), to help cells to cope with cellular stress [82,83]. HIF-1α is overexpressed in PDAC. A homolog of HIF-1α, HIF-2α, has been shown to be expressed at more moderate hypoxic conditions, and stimulates tumour cell proliferation, invasion, and stemness [84]. Downstream effectors of HIF-1α include cyclophilin-a (CypA), chemokine receptor CX3CR1 and Fascins. All promote tumour cell migration and invasion [85,86]. HIF-1α induces the expression of lactate dehydrogenase-a (LDH-A), allowing the tumour cells to generate energy via anaerobic glycolysis [87]. In addition to an increased glycolytic activity, suppression of the production of reactive oxygen species (ROS) is another function of HIF-1α. At moderately increased levels, ROS can aid tumour progression, whereas very high levels of ROS are detrimental to the tumour cells. ROS levels thus need to be kept within a narrow bandwidth [88].

Hypoxia is a mediator of chemoresistance and exerts its effect via several mechanisms. Hypoxia is reported to affect chemosensitivity through Hh pathway upregulation, stimulation of NF-κB signalling, or ERK1/2 signalling and reduction of chemotherapy-induced ROS [89,90,91,92,93]. Radioresistance occurs in hypoxic tumours as the sensitivity of tumour cells to radiation is largely dependent on oxygen [94]. The lack of vasculature also impedes the delivery of drugs, adding an additional layer of chemoresistance. When targeting the ECM, this indirectly allows reconstruction of the tumour vasculature. The characterization of treatments normalizing the vasculature is necessary to completely counteract chemo- and radio-resistance [95].

## 5. Stromal Component: Cancer-Associated Fibroblasts

CAFs are the major cellular component in the PDAC stroma and play a key role in PDAC development and progression [96,97]. CAFs are a heterogeneous population. In mouse models, two distinct populations of CAFs have been identified, marked by either fibroblast specific protein-1 (FSP1) or by α-smooth muscle actin (α-SMA). Mixed populations were observed as well, overlapping with other mesenchymal markers [98,99]. The existence of multiple CAF subpopulations is in part due to multiple precursors that have been identified, and the extrinsic factors that act on these precursors to yield activated stromal cells. CAFs can originate from resident fibroblasts, bone marrow derived cells (BMDCs), and stellate cells [100]. Epithelial cells that have undergone EMT have been proposed to act as precursors as well [96]. All these intrinsic and extrinsic factors lead to different populations that confer considerable heterogeneity to the tumour bulk. In literature, both the terms CAFs and are often used to refer to activated fibroblasts. PSCs are the main source of fibroblasts (CAFs) in PDAC.

### 5.1. Pancreatic Stellate Cells

In the healthy pancreas PSCs comprise 4–7% of the pancreatic parenchymal cells and are characterized by expression of desmin, vimentin, nestin and glial fibrillary acidic protein (GFAP) [101]. The main function in the normal pancreas is the production of pancreatic connective tissue, MMPs and their inhibitors, TIMPs for tissue homeostasis [102]. In cancer, the activation of PSCs is highly dependent on Hh proteins from tumour cells [27,28].

During activation, quiescent PSCs lose their vitamin A lipid droplets and start expressing α-SMA [103]. The activation of PSCs is triggered by cytokines like interleukin-1 (IL-1), IL-6, IL-8, tumour necrosis factor α (TNFα) and growth factors like platelet derived growth factor (PDGF) and TGF-β. These mediators are secreted by damaged cells, tumour cells, and leukocytes [104,105]. Normally, activated PSCs undergo apoptosis when the injury is resolved and normal pancreatic architecture can be restored [106]. However, repeated or sustained injury keeps PSCs activated and the production of excessive amounts of ECM components is sustained, causing the characteristic fibrosis found in PanINs and PDAC [107].

Compelling evidence supports the existence of an essential interaction between PDAC tumour cells and PSCs that results in an increased tumour growth and metastases [108,109]. Tumour cells stimulate PSCs with growth factors and in turn PSCs create a supporting niche for the tumour cells. This activation of PSCs is not uniformly as the secretion of for example HGF concentration, secreted by different populations of PSCs exhibited a wide variation [110]. Activated PSCs promote tumour cells to undergo EMT by reducing the expression of adhesion proteins and stimulating expression of mesenchymal markers [111]. Proteins secreted by PSCs are often associated with migration, invasion and metastases, like WNT2, WNT5a, PLAUR and HGF [110,112,113,114]. The role of PSCs in metastatic dissemination extends beyond dissemination from the primary site, as PSCs are also found at the sites of distant metastases they likely also act in tumour cell intravasation [115]. PDAC tumour cells and PSCs presumably migrate together to distant sites, where PSCs play an important role in seeding and survival of the tumour cells.

In preclinical mouse experiments, targeting the stroma with Hh pathway inhibitors and PDAC tumour cells with chemotherapy enhanced the survival time of mice [116]. Phase III clinical trials applying these stroma targeting agents with first line chemotherapeutics however failed to show these promising results [117]. Later pre-clinical research has shown that when ablating a-SMA positive fibroblasts [118], knocking out SHH ligand [119], or pharmacological inhibition of the Hh pathway [120] increased the aggressive behaviour of tumour cells. This emphasizes the dual function of the stroma; it not only stimulates tumour growth, but also restrains tumour cell proliferation and promotes differentiation.

### 5.2. PSC-Induced Chemoresistance

Activated PSCs play an important role in chemoresistance, either by a mechanical protective role of the PSCs, or through secreted proteins. Addition of PSC-conditioned medium in vitro protects tumour cells from gemcitabine treatment or radiotherapy [97]. This protection can be mediated by the expression of stromal CYR61, which reduces the expression nucleoside transporters hENT1 and hCNT3 in tumour cells to reduce the uptake of gemcitabine as was also shown in vitro [121]. The protective role of the PSCs can also be explained by the expression of cytidine deaminase (CDA) in PSCs, as CDA metabolizes gemcitabine to an inactive form, reducing its effective concentrations [30]. 

As 90% of the tumour bulk can be occupied by the stroma, and this stroma is mainly occupied by PSC-derived CAFs, these cells determine the fate of PDACs tumours to a great extent. An example of this stromal influence is evident from the analysis by Moffitt et al. [9]. Two different stromal subgroups have been described and these strongly associate with clinical outcome.

## 6. Stromal Component: Immune Cells

Immune cells are attracted to inflammatory signals and normally function to protect the body from infectious diseases caused by pathogens. The balance between pro- and anti-tumorigenic factors, produced in the TME, determine the function of infiltrated immune cells [122]. Depending on the cytokines secreted by tumour cells, PSCs, or immune cells already present in the TME, highly diverse populations of immune cells can be attracted. Infiltrated immune cells therefore constitute another important part of the TME, contributing for a great part to inter- and intra-tumour heterogeneity.

### 6.1. Macrophages

Macrophages are essential in tissue homeostasis and defence against foreign pathogens. Resident macrophages secrete a wide range of chemokines and cytokines, triggering immune cell recruitment [123]. Monocytes migrate to the tissue following chemoattractant signals and extravasate from the blood vessels and undergo differentiation into macrophages [124]. Several subpopulation of macrophages have been identified, of which the classically activated macrophages (M1) and Alternatively activated macrophages (M2) are the best documented [125]. M1 macrophages are able to coordinate an effective adaptive immune response against invading pathogens, apoptotic cells or neoplastic cells that disrupt the homeostasis [123]. M2 macrophages display distinct metabolic activities and opposite immune functions when compared with M1 macrophages. M2 macrophages are involved in repairing processes such as wound healing and fibrosis, with the expression of TGF-β as an important mediator of suppression of inflammation [126].

Resident and infiltrated macrophages present within the TME are called tumour-associated macrophages (TAMs) and have been identified as one of the most abundant immune infiltrated cells in PDAC [127]. High amounts of TAMs in the TME are associated with poor prognosis in PDAC [128]. In contrast to the conventional binary M1/M2 definition, TAMs compose an array of subpopulations that share features of both M1 and M2 macrophages [129]. M1-like macrophages are abundant at sites of chronic inflammation where tumours start developing, while macrophages switch to a tumour-promoting M2-like phenotype in established tumours [130]. In a mouse model for PDAC the recruitment of macrophages correlated with an increased expression of TNF-α and MMPs, promoting ECM remodelling [131]. TAMs can promote metastases via the expression of VEGF-A, induce extravasation of tumour cells or induce angiogenesis and immunosuppression via PI3Kγ [132,133].

Therapeutic strategies aiming to reprogram TAMs into an effective anti-tumour activity have started to emerge. Low dose irradiation in xenotransplant mouse models reprogrammed M2-like to M1-like macrophages, which resulted in effector T-cell recruitment [134]. Similarly, in PDAC patients adjuvant gemcitabine-based chemotherapy also modulated macrophage polarization towards M1-like phenotype, activating cytotoxic gene expression programs which correlated with improved clinical outcome [135].

### 6.2. Neutrophils

Neutrophils are another first line of immunity, characterized by their ability to phagocytose, release granules containing lytic enzymes, and to produce ROS [136]. Neutrophils present in the tumour are called tumour-associated neutrophils (TANs) and can be divided into two different subpopulations, either anti-tumorigenic (N1) or pro-tumorigenic (N2) phenotypes. N1 TANs are present in early tumour stages whereas N2 TANs are predominantly found in established tumours. Little data support the anti-tumour function of N1 TANs. What is known is that the N1 TANs are stimulated by IFN-β expression, upon which they induce angiogenesis, and depletion of these N1 TANs impairs cytotoxic T-cell activation [137,138]. When TANs are stimulated with TGF-β, they differentiate towards an N2 state, expressing high levels of MMP-9, arginase and VEGF, factors promoting angiogenesis and facilitating an immune suppressive TME [139]. Neutrophil elastase (NE), a neutrophil secreted protease can modulate tumour cell proliferation and migration by the activation of the proliferative PI3K pathway and cleaving E-cadherin adhesion proteins present on tumour cells [140]. Circulating tumour cells are protected by suppression of peripheral leukocyte activation in the blood stream [141]. This increases the chance of distant metastases, just as the production of neutrophil extracellular traps (NETs). These NETs are able to trap circulating tumour cells and facilitate extravasation to metastatic sites [142].

The neutrophil to lymphocyte ratio (NLR) is a prognostic factor that compares the absolute neutrophil and lymphocyte count pre-operatively in blood samples, wherein a high NLR is associated with poor prognosis [143]. Neutrophils inhibit T lymphocyte activation by expressing arginase, which depletes L-arginine, necessary for activation [144].

### 6.3. Lymphocytes

The second line, or the so-called adaptive immune response is mediated by Lymphocytes. Lymphocytes that infiltrate the tumour are called tumour-infiltrated lymphocytes (TILs). Different subsets of TILs are present in the TME. Th1 and cytotoxic T cell functionality are generally impaired whereas the function of Th2 and Treg cells are enhanced. In accordance with this, PDAC patients with high Th1 and cytotoxic T cell counts have a good prognosis [145]. Tumour cells prevent the infiltration of these anti-tumour lymphocytes, as the juxtastroma hampers the functionality of cytotoxic T cells [146]. In concordance with this inhibition, computational models of intratumoral T-cells show that high levels of cytotoxic T-cells in proximity to tumour cells are associated with increased survival [147]. Another restriction on the immune system in the TME is the secretion by activated PSCs of IL13, an immunosuppressive protein, inhibiting Th1 while promoting an Th2 response [148].

TIL differentiation into Tregs is regulated by immunosuppressive ligands, mainly expressed by activated PSCs, like TGF-β, IL-10 and IL-2 [149]. Tumour expressed 2,3-dioxygenase differentiates TIL into Tregs as well. Treg are mainly present in the juxtastromal compartment to help the tumour cells evade the immune system [150]. Closely related to the function of Tregs are the Th17 lymphocytes, high levels of TGF-β and IL6 allow differentiation into Th17 [151]. Higher frequencies of Th17 cells promote angiogenesis and lymph nodes metastases, but they are also correlated with increased cytotoxic T cell populations, improving survival [152,153].

Studies identifying prognostic markers have found many markers for immune system components that positively correlate with survival. In contrast, high macrophage infiltration, especially Th2 type macrophage infiltrations, are associated with a worse outcome [154]. These correlations are good for potential therapy development, but the factors that drive these different underlying immunogenic responses should be identified.

## 7. Molecular Subtypes in PDAC

Our understanding of the biology that underlies the growth and progression of tumours has benefited enormously from the identification of gene expression-based subtypes, determined by both supervised and unsupervised classification methods. A large number of studies have employed supervised clustering to classify gene expression profiles using clinical parameters such as survival and metastatic propensity [155,156,157]. Unsupervised clustering does not rely on known data or parameters to identify groups, but rather makes use of fully unbiased sample separation algorithms like non-negative matrix factorization (NMF) and principal component analyses (PCA) [158]. As unsupervised clustering is arguably better suited to uncover previously unrecognized tumour biology compared to supervised methods, we will focus on those efforts here, briefly summarized in Figure 1.

### 7.1. Collisson 2011 

As the first to classify PDAC into gene expression based subtypes, Collisson et al. were hindered by the paucity of pancreatic tissue available for sequencing (*n* = 27) [33]. This was overcome by adding expression data from human and mouse cell lines (*n* = 34) and by combining the transcription profiles from another study (*n* = 36). Three prognostic subtypes were identified: classical, quasi-mesenchymal (QM) and exocrine-like, that could be identified by a classifier called PDAssigner. The classical subtype is one that is defined by high expression of adhesion specific and epithelial genes and has the best survival. The QM subtype showed higher expression of mesenchymal genes, and was associated with the poorest prognosis. The exocrine subtype was associated with the expression of digestive enzyme related genes. This subtype however was not found in cancer cell lines, and this has led to some discussion on how tumour-specific the exocrine subtype is.

### 7.2. Kim 2013 

This classification of 96 PDAC samples resulted in three gene expression-based subtypes [159]. The subtypes were differentiated by survival, where type 1 and 2 were significantly different (median OS 37.6 vs. 19.2; *p* = 0.001). The molecular gene signatures were compared to the PDAssigner from Collisson et al. and subtype 3 had similar defining differentially expressed genes as the exocrine subtype, arguing in favour of the existence of the exocrine subtype. No comparisons were shown to correlate type 1 and 2 subtypes to the PDAssigner.

### 7.3. Moffitt 2015 

To delineate the tumour-specific gene expression in PDAC, Moffitt and colleagues incorporated primary tumour, metastatic tumour and normal samples [9]. By performing NMF with high cluster numbers, specific gene expression patterns were identified that were allocated to normal pancreas expression and weighed as such in further analyses. This resulted in the identification of two tumour subtypes: A classical subtype that resembled the classical group from Collisson et al., with hallmark genes that largely overlapped. A basal subtype identified poor-prognosis patients. In addition, specific stromal gene expression was dissected and this was used to define two stromal subtypes; normal and activated. The activated stroma subtype was characterized by a diverse set of genes associated with macrophages and chemokine ligands, leading to the hypothesis that the activated stroma is characterized by a more activated inflammatory signature. Basal and epithelial tumours were both found in activated and normal stroma, wherein the activated stroma subtypes added to a worse prognosis.

### 7.4. Gutiérrez 2015 

Using a set of 27 PDAC patients, two distinct GEPs (Gene Expression Profiles) were identified via PCA and hierarchical clustering [160]. GEP-A was characterized by an overexpression of genes associated with cellular stress, injury response and chronic inflammatory diseases. GEP-B tumours were less altered, in terms of gene expression compared to GEP-A tumour samples, but were characterized by a decreased expression of cell junction and intercellular adhesion genes and an increased expression of immunosuppressant pathways. Correlating these classes to the subtypes of Collisson et al. showed that the GEP-A subtype associated with the classical subtype and GEP-B tumours most closely related to the QM subtype.

### 7.5. Janky 2016 

For this study 118 tumour PDAC samples and 13 normal pancreas samples were sequenced [161]. The set was clustered using the PDAssigner, as well as by NMF. The number of clusters (k) was set to 2, 3, 4 or 5 and when these clusters were compared to the PDAssigner there was a 92.4% overlap with their subtypes in the NMF clustering using three clusters. The classical subtype of Collisson et al. correlated to k3.cl1 and to the best survival. The exocrine subtype, k3.cl2 was associated with the worst survival, in contrast to what Collisson et al. found. The mesenchymal subtype, k3.cl3 had comparable survival to the k3.cl2.

### 7.6. Bailey 2016 

Expression analysis on 96 tumours was performed by RNA-Seq in addition to the genomic analyses on 456 samples [32]. Unsupervised clustering revealed four subgroups. These were named pancreatic progenitor, aberrantly differentiated endocrine exocrine (ADEX), squamous, and immunogenic. Gene programs were used to describe the distinct biological processes that underlie the four subgroups. The pancreatic progenitor (related to the classical subtype) featured few upregulated gene expression patterns, but was characterized by networks enriched for transcription factors like PDX1, HNFs and FOXA2/3, indicating deregulations in transcription regulation. The ADEX (related to the exocrine subtype), was characterized by exocrine and β cell development gene expression patterns. The squamous subtype was defined by an activated state of inflammation- and hypoxia- gene expression, and genes upregulated in ECM, TGF-β and WNT signalling. A novel subtype was the immunogenic subtype, distinguished by expression patterns from the adaptive immune system like B-cell and CD-4 and -8 positive T-cell expression. The innate immune system was associated with the squamous subtype. The immunogenic subtype had a better overall survival compared to the worst prognosis subgroup, suggesting that the involvement of the adaptive immune response is beneficial. 

### 7.7. The Cancer Genome Atlas (TCGA) Research Network 2017 

Combined genomic, transcriptomic and proteomic analyses of 150 PDAC patient samples were performed with whole exome sequencing (WES), RNAseq, miRNAseq, DNA methylation and reverse-phase protein array (RPPA) [20]. Genomic analyses were descriptive for the known driver mutations and exploratory on other mutations that could be targeted genotype-specifically. The transcriptomic and proteomic analyses were hampered by a high number of samples with low tumour percentage. Previous studies performed pre-selection of whole tumour samples by selecting only samples with a tumour content of 30–40% or higher, or performed macro- or micro-dissection to obtain higher tumour cellularity in the samples. Not wanting to lose the information on the low cellularity samples, the 150 samples were dichotomized on tumour cellularity obtaining two groups (≥33% and <33%). After this segregation unsupervised class discovery was performed. The high tumour cellularity samples correlated with the squamous and progenitor subtype of Bailey et al. [32] and the classical and quasi-mesenchymal of Collisson et al. [33]. The exocrine and immunogenic subtypes had a higher correlation to the low tumour cellularity subgroup, indicating a higher influence of other non-tumour cellular expression patterns to these subtypes. No new subtypes were generated. The other analyses, on DNA methylation, lnRNA, miRNA and protein expression were performed on the high tumour cellularity set, since classifications on the low purity samples were not feasible.

## 8. Discussion

**Figure 2:**
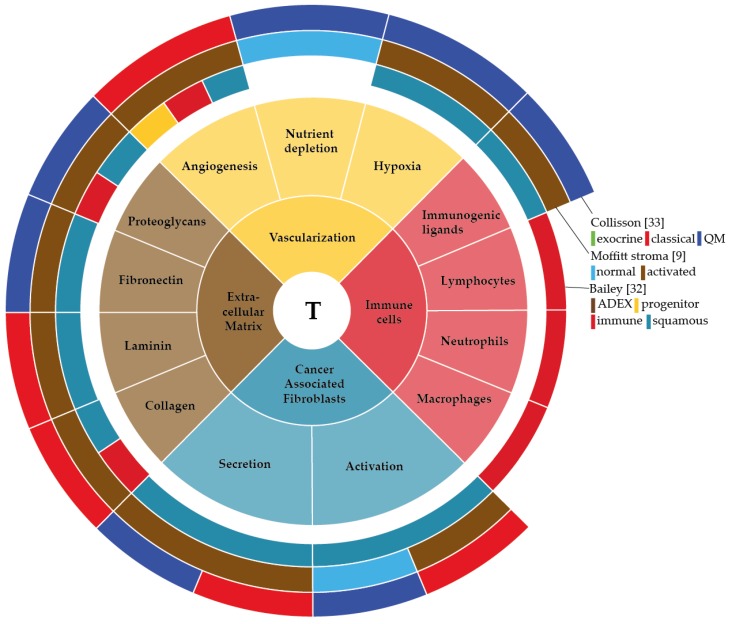
Schematic representation of the stromal elements described in this review that contribute to, or interconnect with subtype-specific gene expression in PDAC (outer circle, Collisson [33]; middle circle, Moffitt stroma [9]; inner circle, Bailey [32]). Middle circle represents the tumor (T), surrounding the tumor is the stroma, representing the sections and subsections. Proteins described in this review were checked for expression in the subtypes as summarized in the figure. Colours are based on the colours coding used in the original publications.

As apparent from the classification described by Moffitt et al. the stroma in PDAC can show substantial differences in activation status and contribute significantly to bulk tumour heterogeneity and patient outcome. In this review, we have given an overview of the non-tumour cell populations that contribute to intertumour heterogeneity in bulk PDAC tissue, and summarized this in Figure 2. This leaves a central question unanswered; how exactly do these stromal cells contribute to gene expression-based heterogeneity at the bulk tumour level and influence patient outcome?

We hypothesize that the stromal influences on bulk tumour heterogeneity are based on three principles: (1) A differential composition of the stroma, in which “the stroma” is just the sum of the gene expression of these individual components. For example, a relatively high fraction of immune cells infiltrated in a tumour will yield an “immune” subtype tumour, whereas a paucity of (myo)fibroblasts is likely to result in a “classical” or canonical/epithelial subtype. As the tumour host and thus the stroma are genetically homogenous, a differential cellular composition of the stroma is likely to be the result of differential instruction from the genetically instable tumour cells. One observation that argues against this is that Moffitt’s activated stroma subtype does not specifically associate with the classical or basal subtype. (2) It is also plausible that within defined stromal cell populations, such as the fibroblasts, the crosstalk with tumour cells has yielded a very distinct gene expression pattern for that population of cells that is as heterogeneous between patients as the tumour cell compartment is. Again, this heterogeneity likely originates from early events in the tumour cells that impact on the stromal cells (for instance a SMAD4 mutation [162]), but it is also possible that stochastic or epigenetic events in the stromal gene expression pattern drive this. (3) A combination of the above. This is the likely scenario in which the heterogeneity in the individual stromal cell populations as well as differences in the cellular composition are combined. This could yield highly demarcated stromal subtypes with distinct biological programs dominating, but it can also be argued that this complexity comes with an amount of “noise” that precludes the identification of separate subgroups. This could be the reason why Moffitt’s classification resulted in only two stromal subgroups based on activation status, and why others have described differential secretion of HGF by PSCs as “heterogeneity” [110]. In any case, it is now clear that whatever the cause of stromal heterogeneity may be, it impacts on the tumour cells and the tumour as a whole.

From the above, a “chicken or the egg” causality dilemma is apparent. Do the genetically driver tumour cells first instruct their surroundings, attracting their own specific TME, and thus determine their own fate? This would mean that the TME initially has a low heterogeneity and that during the development of the tumour, the heterogeneity of the TME evolves along. Or is the establishment of the TME stochastically coordinated, in other words; the TME largely determines its own fate by chance, and subsequently of the tumour cells as well. If a complex crosstalk loop is indeed required throughout tumour progression, stochastic events in the stroma could be given the chance to exert long-lasting and significant effects. The complex interactions between stromal cell populations allow and support such cross talk. For instance, an additional layer of complexity in the stroma comes from the differential regulation of vascularization under the influence of PSCs, which are instructed by tumour cells. In turn, these effects on vascularization are likely to impact on immune infiltration further adding to heterogeneity.

A more daring hypothesis is that the development of the stroma is largely uncoupled from the tumour compartment, and that the interactions within the stroma are sufficient to generate heterogeneity that is measurable at the whole tumour level. Along those lines, more systemic patient characteristics could initiate stroma heterogeneity; for instance, if a given patient has high numbers of circulating mesenchymal stem cells this could result in a very fibroblast-rich tumour that will likely classify as “mesenchymal”. A similar scenario could be envisioned for immune cells and the resulting tumour bulk subtype. Experimental models that allow longitudinal assessment of intertumour heterogeneity from onset (PanIN) to late stage disease to test these hypotheses should yield much needed insight.

## 9. Conclusions

Concluding, the classification of PDAC at the bulk tumor gene expression level holds promise for clinical decision-making but this will take time and rigorous validation. Perhaps it could be argued that, given the important contributions of stromal cells to these classifications and also the abundance of this tissue in PDAC, we should investigate treatments that specifically consider these stromal contributions to tumor biology, and importantly, the heterogeneity that exists in this compartment. This notion is supported by the advent of clinical trials using stroma targeting agents (Table 1), but there are few reports on positive results from such trials. Patient stratification using knowledge on stromal heterogeneity could improve outcomes.

## Figures and Tables

**Table 1:** Overview of current active clinical trials targeting the TME. Studies were identified on clinicaltrials.gov.

Start.	Trial	No Pts	Study Design	Treatment	TME Targeting Agent	PI
2017	NCT03307148	34	Single group assignment	ATRA with Gemcitabine and Nab-paclitaxel	ATRA: all *trans* retinoic acid, involved in the inactivation of PSCs	David Propper
2017	NCT03098160	69	Sequential assignment	Evofosfamide and ipilimumab	Evofosfamide: hypoxia-directed cytotoxic agent Ipilimumab: activating cytotoxic T-cells	David Hong
2017	NCT03168139	20	Single group assignment	Olaptesed pegol w/wo Pembrolizumab	Olaptesed: targeting CXCL12, involved in vascular homeostasis Pembrolizumab: inhibition of PD-1 receptor, immune checkpoint inhibitor	Unknown
2017	NCT03277209	28	Single group assignment	Dose escalation plerixafor	Plerixafor: targeting CXCR4, receptor for stroma derived factor-1, involved modulation of the immune micro-environment	Elizabeta Popa
2016	NCT02777710	58	Single group assignment	Durvalumab and Pexidartinib	Durvalumab: inhibits PD-L1, immune checkpoint inhibitor Pexidartinib: inhibits CSF1R, depleting the TME from M2-like TAMs	Philippe Cassier
2106	NCT02921022	56	Non-randomized parallel assignment	Prophylactic/therapeutic rivaroxaban with Gemcitabine, Nab-paclitaxel and PEGPH20	PEGPH20: hyaluronidase enzyme, targeting HA	Kenneth Yu
2016	NCT02726854	30	Single group assignment	Apatinib	Apatinib: VEGFR-2 inhibitor, inhibiting angiogenesis	Enxiao Li
2015	NCT02451982	50	Randomized parallel assignment	Cyclophosphamide and GVAX Pancreatic cancer w/wo Nivolumab	GVAX: vaccine secreting granulocyte-macrophage colony stimulating factor, stimulates immune response Nivolumab: inhibits PD-L1, immune checkpoint inhibitor	Lei Zheng
2015	NCT02546531	50	Non-randomized parallel assignment	Dose escalation/expansion of defactinib, pembrolizumab and gemcitabine	Defactinib: FAK inhibitor, involved in the inhibition of fibrosis and inflammation and anti-tumor effects Pembrolizumab: inhibition of PD-1 receptor, immune checkpoint inhibitor	Andrea Wang-Gillam
2015	NCT02179970	28	Single group assignment	dose escalation plerixafor	Plerixafor: targeting CXCR4, receptor for stroma derived factor-1, involved modulation of the immune microenvironment	Duncan Jodrell
2014	NCT02030860	15	Randomized single group assignment	Gemcitabine, Nab-paclitaxel w/wo Paricalcitol	Paricalcitol: active vitamin D2 analog, targeting fibrosis in the TME	Peter O’Dwyer
2014	NCT02159989	69	Single group assignment	Sapanisertib and Ziv-Aflibercept	Ziv-Aflibercept: VEGF inhibitor, involved in angiogenesis inhibition	Aung Naing

## Abbreviations

PDAC	Pancreatic ductal adenocarcinoma
PC	Pancreatic cancer
CAFs	Cancer associated fibroblasts
PSCs	Pancreatic stellate cells
ECM	Extracellular matrix
MCN	Mucinous cystic neoplasms
IPMN	Intraductal papillary mucinous neoplasms
PanIN	Pancreatic intraepithelial neoplasia
KRAS	KRAS proto-oncogene
CDKN2A	Cyclin dependent kinase inhibitor 2A
TP53	Tumor protein 53
SMAD4	SMAD family member 4
CNV	Copy number variation
ERBB2	Erb-b2 receptor tyrosine kinase 2
MET	MET proto-oncogene, receptor tyrosine kinase
CDK6	Cyclin dependent kinase 6
PIK3CA	Phosphatidylinositol-4,5-biphosphate 3-kinase catalytic subunit alpha
PIK3R3	Phosphoinositide-3-kinase regulatory subunit 3
BRCA	Breast cancer (susceptibility gene)
PALB2	Partner and localizer of BRCA2
Hh	Hedgehog
TGF-β	Transcription Growth Factor β
SMAD4	SMAD family member 4
MAPK	Mitogen associated protein kinase
TME	Tumor microEnvironment
MMPs	Matrix metalloProteases
TIMPs	Tissue inhibitor metalloproteases
EGF	Epidermal growth factor
FAK	Focal adhesion kinase
DDR2	Discoidin domain receptor tyrosine kinase 2
NF-κβ	Nuclear factor kappa beta
EMT	Epithelial-to-mesenchymal transition
SHH	Sonic Hedgehog
SPOCK	Sparc/osteonectin, cwcv and kazal-like domains proteoglycan
HA	Hyaluronic acid
CD44	Cluster of differentiation 44
RHAMM	Receptor for HA mediated motility
CAM-DR	Cell adhesion mediated drug resistance
ROCK	Rho-associated protein kinase
ECs	Endothelial cells
vSMCs	Vascular smooth muscle cells
VEGF	Vascular endothelial growth factor
bFGF	basic fibroblast growth factor
HIF1α	Hypoxia inducible factor 1α
CypA	Cyclophilin A
LDH-A	Lactate dehydrogenase A
ROS	Reactive oxygen species
TCA cycle	Tricarboxylic acid cycle
FSP1	Fibroblast specific protein 1
α-SMA	α-Smooth muscle actin
BMDCs	Bone marrow derived cells
GFAP	Glial fibrillary acidic protein
IL	Interleukin
TNFα	Tumor necrosis factor α
PDGF	Platelet derived growth factor
TAM	Tumor associated macrophage
NE	Neutrophil elastase
NET	Neutrophil Extracellular Traps
NLR	Neutrophil to lymphocyte Ratio
TIL	Tumor infiltrating lymphocytes
NMF	non-negative matrix factorization
QM	Quasi mesenchymal
GEP	Gene expression profiles
ADEX	Aberrantly differentiated endocrine exocrine
TCGA	The Cancer Genome Atlas
